# Refinement of the novel tank diving test: toward standardized and robust analysis of anxiety-like behavior in zebrafish

**DOI:** 10.3389/fnbeh.2025.1624277

**Published:** 2025-10-29

**Authors:** Takuro Shinkai, Misa Nakai, Uzuki Takeshita, Kento Morita, Yasuhito Shimada

**Affiliations:** ^1^Graduate School of Regional Innovation Studies, Mie University, Tsu, Japan; ^2^Mie University Zebrafish Research Center, Tsu, Japan; ^3^National Museum of Ethnology, Suita, Osaka, Japan; ^4^Lake Biwa Museum, Kusatsu, Japan; ^5^Graduate School of Bioresources, Mie University, Tsu, Japan; ^6^Graduate School of Engineering, Mie University, Tsu, Japan; ^7^Zebra Innovate LLC, Tsu, Japan; ^8^Graduate School of Medicine, Mie University, Tsu, Japan

**Keywords:** drug testing, environment, *in vivo*, model animal, quantitative analysis, stochastic model

## Abstract

The novel tank diving test (NTT) is a widely used behavioral assay for evaluating anxiety-like behaviors in zebrafish; however, results often exhibit considerable variability across different experimental settings. In this study, we systematically analyzed various methodological factors influencing the outcomes of NTT and introduced refinements to enhance its reliability and reproducibility. We optimized the detection parameters for region entry and freezing behavior using logistic regression analysis, significantly reducing false-positive classifications caused by tracking artifacts. The impact of pre-test stress conditions—restraint and darkness—was assessed, demonstrating that restraint effectively decreased the variability in behavioral parameters, such as latency to enter the top half (LTTH) of the tank and frequency of entries (FE). Conversely, combining darkness with restraint induced abnormal behaviors, limiting utility of the test. The effects of temperature were also rigorously evaluated, revealing that even subtle deviations within 3 °C of the standard temperature of 26.5 °C significantly affected behavioral variability, and 26.5 °C was optimal for reliable outcomes. Furthermore, we demonstrated that net-chasing during fish handling significantly increased the freezing time, suggesting the adoption of funnel-based transfers to reduce stress artifacts. Finally, behavioral patterns during stable test conditions followed a Poisson process, enabling the estimation of optimal test durations. Overall, our proposed refinements help establish a standardized, robust NTT protocol that minimizes variability and enhances the assay’s sensitivity and reproducibility to investigate anxiety behavior in zebrafish.

## 1 Introduction

Over the past two decades, zebrafish have been utilized as a model animal for various human diseases. They have been actively employed in a broad range of studies related to the central nervous system. These include microscopic examinations, such as the visualization of intracellular calcium concentrations in neurons, as well as macroscopic neurobehavioral analyses, which encompass learning abilities (Ahrens et al., 2013; Chen et al., 2013; Marvin et al., 2013), anxiety behaviors (Egan et al., 2009; Cachat et al., 2010; Maximino et al., 2010), and group (schooling) behaviors (Miller and Gerlai, 2012). Additionally, zebrafish are commonly used as models for various diseases, such as Parkinson’s disease (Bretaud et al., 2004), Alzheimer’s disease (Saleem and Kannan, 2018), amyotrophic lateral sclerosis (Van Hoecke et al., 2012; Ciura et al., 2013), and several types of epilepsy (Berghmans et al., 2007; Baraban et al., 2013). Anxiety disorders are closely related to diseases of the central nervous system. This is because various parts of the nervous system can affect each other, and one disease can lead to dysfunction in other parts of the nervous system. For example, depression and anxiety disorders often coexist owing to a common mechanism of imbalance in neurotransmitters, such as serotonin, norepinephrine, and dopamine (Malhi and Mann, 2018). Moreover, in Parkinson’s disease, a decrease in dopamine can readily induce anxiety (Armstrong and Okun, 2020), and anxiety disorders are also common in Alzheimer’s disease (Cummings and Kaufer, 1996).

For investigating anxiety behaviors using zebrafish, various methods are employed, including the novel tank diving test (also known as the novel tank test; NTT) (Bencan et al., 2009; Stewart et al., 2011; Blaser and Rosemberg, 2012), the light and dark test (scototaxis) (Maximino et al., 2011, 2012), tapping assay (Ro et al., 2022), and acute exposure to conspecific alarm substances (Speedie and Gerlai, 2008). Among these, many scholars have supported that NTT is highly reliable and relatively easy to conduct; thus, it is one of the most employed assays in zebrafish testing. Notably, NTT has been used as an evaluation system for the anti-anxiety effects of various compounds, including nicotine (Duarte et al., 2019), naturally derived ingredients (Abidar et al., 2020), and gut bacteria, such as lactobacilli (Davis et al., 2016; Schneider et al., 2016; Olorocisimo et al., 2023).

In the NTT, zebrafish initially remain near the bottom of the tank upon introduction to a new environment, a behavior commonly interpreted as a response to potential threat or novelty. Over time, the fish begin to explore upper regions of the tank, which is considered to reflect a reduction in anxiety-like behavior rather than acute fear. In practice, the fish are placed in a narrow tank, and researchers observe and record only their vertical movements. Although this assay may appear relatively simple, the results are influenced by various internal and external factors. Variables, such as the size (height) of the tank, duration of observation, and level of pre-stress experienced by the fish, differ among studies, leading to considerable variability in the results of NTT (Anwer et al., 2021; Muralidharan et al., 2024). To address this issue, many studies have increased the sample size. Zebrafish offer an advantage over mice as they are easier to handle and allow for the formation of larger groups. Although *in vivo* tests are generally prone to variability, if experimental conditions can be established to reduce this variability even with smaller sample sizes, the significance of NTT as a zebrafish-based testing method will further increase.

In this study, we analyzed the factors influencing the test results of NTT and established an assay protocol with reduced deviation. Furthermore, we proposed a new approach for quantifying anxiety levels.

## 2 Materials and methods

### 2.1 Zebrafish

Wild-type zebrafish (*Danio rerio*; AB line) were originally obtained from the Zebrafish International Resource Center (Eugene, OR, US) and subsequently bred and maintained in our laboratory under standard conditions (28 ± 0.5 °C, 14/10 h light/dark cycle). In some experiments, we individually identified the fish using visible implant elastomer tags (Tanaka Sanjiro, Fukuoka, Japan), which is made of silicon and contains fluorescent dyes (Frederickson et al., 2023). The experimental procedures were conducted in accordance with the Japanese Animal Welfare Regulatory Practice Act on Welfare and Management of Animals (Ministry of the Environment of Japan) and in compliance with the guidelines of Animal Research: Reporting of *In Vivo* Experiments (ARRIVE). Ethical approval from the local Institutional Animal Care and Use Committee was not sought because this law does not mandate the protection of zebrafish.

### 2.2 Novel tank diving test (NTT)

We typically maintained 10–20 fish in the breeding system. A few days before testing, the fish were moved into separate 2-L tanks with five fish per tank to equalize social conditions. This configuration was chosen to reduce the stress associated with net chasing during test fish collection, which can increase anxiety-related behavior such as freezing time. Additionally, an international multi-laboratory study recommended a housing density of 5 fish per liter as optimal for the NTT (Hillman et al., 2025). To assess the effects of experimental conditions while minimizing individual variation, all tests were conducted using the same group of adult zebrafish (6–9 months). An equal number of males and females were included, and individuals that were noticeably undersized or oversized were excluded prior to testing.

The NTT protocol is divided into acclimation, pre-test stress loading, and testing. In the acclimation step, fish were transferred into a 600-mL tank (Meito-Suien, Aichi, Japan; internal dimensions: 135 mm (width) × 95 mm (diameter) × 90 mm (height)) for 5 min, after which they were transferred to the holding tank for an additional 5 min (pre-test stress loading, as described below). The fish were subsequently transferred to the test tank for NTT. The test tank’s internal dimensions are as follows: width, diameter, and height of 140, 45, and 120 mm, respectively. Its front wall is composed of 2-mm transparent acrylic, and its sides and bottom are composed of 5-mm white acrylic. All side walls except for the front comprise two overlapping acrylic panels (thicknesses of 2 and 3 mm) to minimize heat loss. The acrylic panels were bonded using an acrylic adhesive (Acrysunday, Tokyo, Japan), a solvent-based adhesive commonly used in aquarium construction. The tanks were rinsed multiple times before use to ensure biological and chemical safety for aquatic animals. Trial duration was 15 min and recordings were performed in an isolated and sound controlled room. We filmed the experimental arena from a horizontal angle at 30 frames per second (fps) using a digital high-definition (HD) video camera (Panasonic HC-V495M, Osaka, Japan).

To prevent heat loss, windows were covered with double layers of bubble wrap, all gaps were sealed, and blackout sheets were used to completely block external light. An air conditioner and a circulation fan were employed to maintain stable room temperature (within ± 1 °C). By combining a reptile panel heater with low-conductivity materials, such as expanded polystyrene board, we achieved water-temperature control to within ± 0.3 °C. Once filming began, the room was left unoccupied to maintain a quiet environment.

Supplementary Methods provide detailed descriptions of the various conditions tested and the corresponding protocols regarding water temperature handling, comparison of pre-test stressors, and distinction of the net-chasing effect.

### 2.3 Pre-test stressors

**FIGURE 1:**
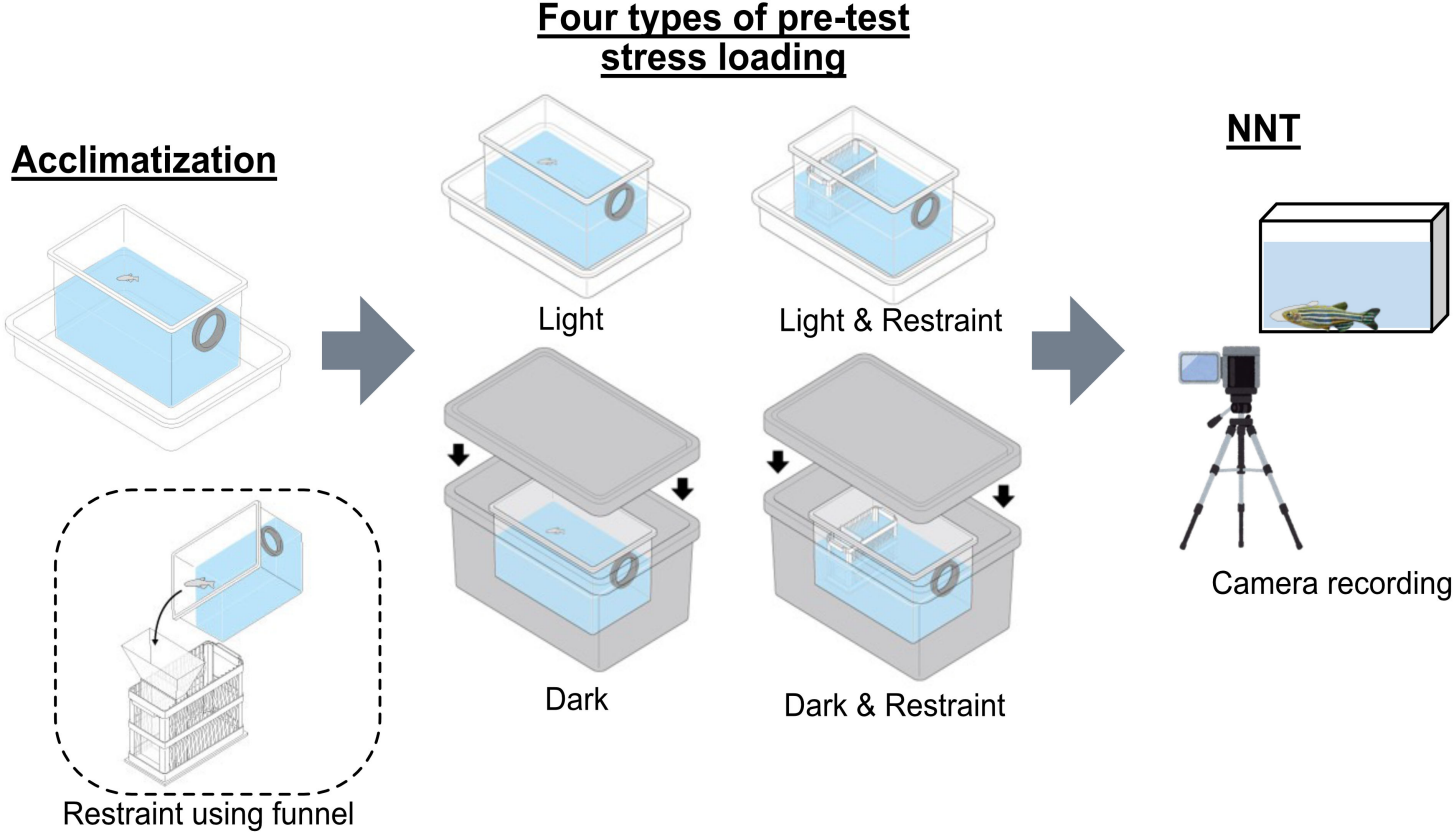
Experimental design of the pre-test stress-loading conditions. A schematic representation of the four pre-test stress-loading conditions by combining the presence or absence of restraint with light or dark environments. Fish were subjected to either restraint or no-restraint and placed under light or dark conditions prior to the novel tank diving test (NTT).

After the acclimation step in NTT, we applied pre-test stress loading that combined light/dark conditions with restraint stress. Using a slide-glass chamber as a base, we fabricated a 4-mm-wide restraint device that prevented the fish from turning, and we produced a matching funnel using a 3D printer. The bright environment was set at 300 lux with illumination provided from the top using a fluorescent tube (Haghani et al., 2019), and the dark environment was created by placing the fish and their tanks inside a light-proof box. Subsequently, we combined the presence or absence of restraint with light or dark conditions to evaluate four types of pre-test stress loading (Figure 1). Total handling time before the main test was kept under 10 min across all groups. To address potential order effects, we employed a randomized block design in which all fish underwent all four conditions (light/restraint, dark/no-restraint, etc.) in different sequences. Additionally, to minimize carryover effects between tests, a 3-day interval was maintained between trials for each individual.

### 2.4 Video analysis

Video files recorded in MTS format were converted to AVI using XMedia Recode 64-bit (version 3.6.0.8). We subsequently tracked each fish’s position with idTracker (Pérez-Escudero et al., 2014; Romero-Ferrero et al., 2019), extracting the movement trajectories [x(t), y(t)] at 1/30-s intervals. The converted videos were reviewed in idPlayer to visually confirm accurate tracking. Any untracked segments were retracked, and obvious movements, such as a fish turning, were used to fill in missing data. For all other gaps, we assumed straight-line motion and conducted linear interpolation by dividing the coordinate difference between the two known endpoints by the number of missing frames. The start time of measurement (*t* = 0) was defined as the moment when the fish came closest to the bottom of the tank after transfer. Using ImageJ (version 1.54, National Institute of Health, Bethesda, MD, USA), we measured the width and height of the tank to determine the pixel-to-millimeter ratio. Based on the coordinates of the water surface and the left edge of the tank, we adjusted the position of the tank within the video frame and corrected the trajectory so that the center of the tank was set as the origin. According to a previous study (Audira et al., 2021), we adopted the following four NTT parameters.

1. Latency to enter the top half (LTTH): time until the fish initially enters the upper half of the tank.
2. Time spent in the top half (TSTH): time spent by the fish in the upper half of the tank during the measurement period.
3. Frequency of entries (FE): number of times the fish enters the upper half of the tank during the measurement period.
4. Freezing time (FT): duration of immobility (speed ≤ 10 mm/s).

### 2.5 Correction formula for behavioral quantification

Machine-based judgments, determined by simple classification of trajectory values based on the defined criteria for LTTH, TSTH, FE, and FT, showed a non-negligible discrepancy from human visual assessments, particularly in detecting entries into designated regions and estimating freezing behavior. To address this, we extracted and compiled specific segments from experimental datasets obtained under various conditions. To enable accurate classification without relying on human visual assessment, we developed custom macro programs in Microsoft Excel (Professional, 2023) and established both threshold adjustments and regression models.

We evaluated false negatives and false positives by comparing judgments made by experienced experimenters with those generated by the software, using the F-score as the performance metric. The F-score is calculated as follows:


$$F-score\;\left(\%\right)=\frac{2\cdot P\,r\,e\,c\,i\,s\,i\,o\,n\cdot R\,e\,c\,a\,l\,l}{P\,r\,e\,c\,i\,s\,i\,o\,n+R\,e\,c\,a\,l\,l}\times 100$$

$$P\,r\,e\,c\,i\,s\,i\,o\,n=\frac{Truepositive\;}{T\,r\,u\,e\,p\,o\,s\,i\,t\,i\,v\,e\,s+F\,a\,l\,s\,e\,p\,o\,s\,i\,t\,i\,v\,e\,s}$$

$$R\,e\,c\,a\,l\,l=\frac{Truepositive\;}{T\,r\,u\,e\,p\,o\,s\,i\,t\,i\,v\,e\,s+F\,a\,l\,s\,e\,n\,e\,g\,a\,t\,i\,v\,e\,s}$$

The F-score ranges from 0% (no precision or recall) to 100% (perfect precision and recall).

For the FE correction formula, we addressed the issue of excessive false positives in region entries caused by a threshold that was too low to define event occurrence. Specifically, we varied the minimum duration required to define an entry from 0 to 5 s (0–150 frames) and calculated the corresponding F-scores. This analysis was conducted using 82,887 frames collected from seven females and 98,420 frames from seven male zebrafish.

Freezing behavior was analyzed using 10,488 frames collected from one male and one female, while swimming behavior was analyzed using 3,217 frames collected from three different males and one female. To detect freezing behavior, we adopted a threshold of 10 mm/s in line with previous zebrafish behavioral study (Audira et al., 2021). While more stringent thresholds such as < 3 mm/s may reduce false positives, they also risk omitting brief freezing-like episodes, which increases false negatives. The 10 mm/s criterion thus represents a practical balance between sensitivity and specificity in detecting immobility in zebrafish. Misclassification of freezing behavior primarily results from mechanical fluctuations during tracking. Therefore, rather than merely adjusting threshold values, it is crucial to eliminate noise. We calculated various parameters—such as the total, mean, and variability of displacement, velocity, and acceleration—and applied both Mahalanobis distance–based discriminant analysis and logistic regression. The performance of each model was evaluated using the F-score, with careful consideration of potential multicollinearity and overfitting, to identify the best-performing configuration.

### 2.6 Statistical analysis

An F-test was conducted for all group comparisons. For fish individually identified using elastomer tags, paired *t*-tests were conducted. In experiments without individual identification, either Student’s or Welch’s *t*-test was applied based on the outcome of the F-test. These analyses were conducted using Microsoft Excel. For comparisons among multiple groups in which the same individuals were tested repeatedly, a one-way analysis of variance (ANOVA) was conducted by linking data through individual identifiers. When a significant main effect was found, *post hoc* comparisons were performed using Tukey’s multiple comparison test. These analyses were performed using Prism 10 (GraphPad Software, San Diego, CA, USA). To assess the cumulative effects of net-chasing, time-series event occurrence data were analyzed using the log-rank test in Prism 10. The index evaluating the validity of the logistic regression model was calculated using SPSS version 27 (IBM, Armonk, NY, USA).

### 2.7 Stochastic model (Poisson process)

The Poisson process is a stochastic model that describes the number of events randomly occurring within a fixed interval of time or space. It is characterized by three key properties: (1) Independence: events occurring in different intervals are independent. (2) Constant rate: the probability of observing κ events in a time interval *t* follows a Poisson distribution with mean λt, where λ is the average event rate per unit time. (3) Memorylessness: the time between successive events follows an exponential distribution, and the probability of future events is independent of past occurrences.

## 3 Results

### 3.1 Optimizing region-entry and freezing time detection

**FIGURE 2:**
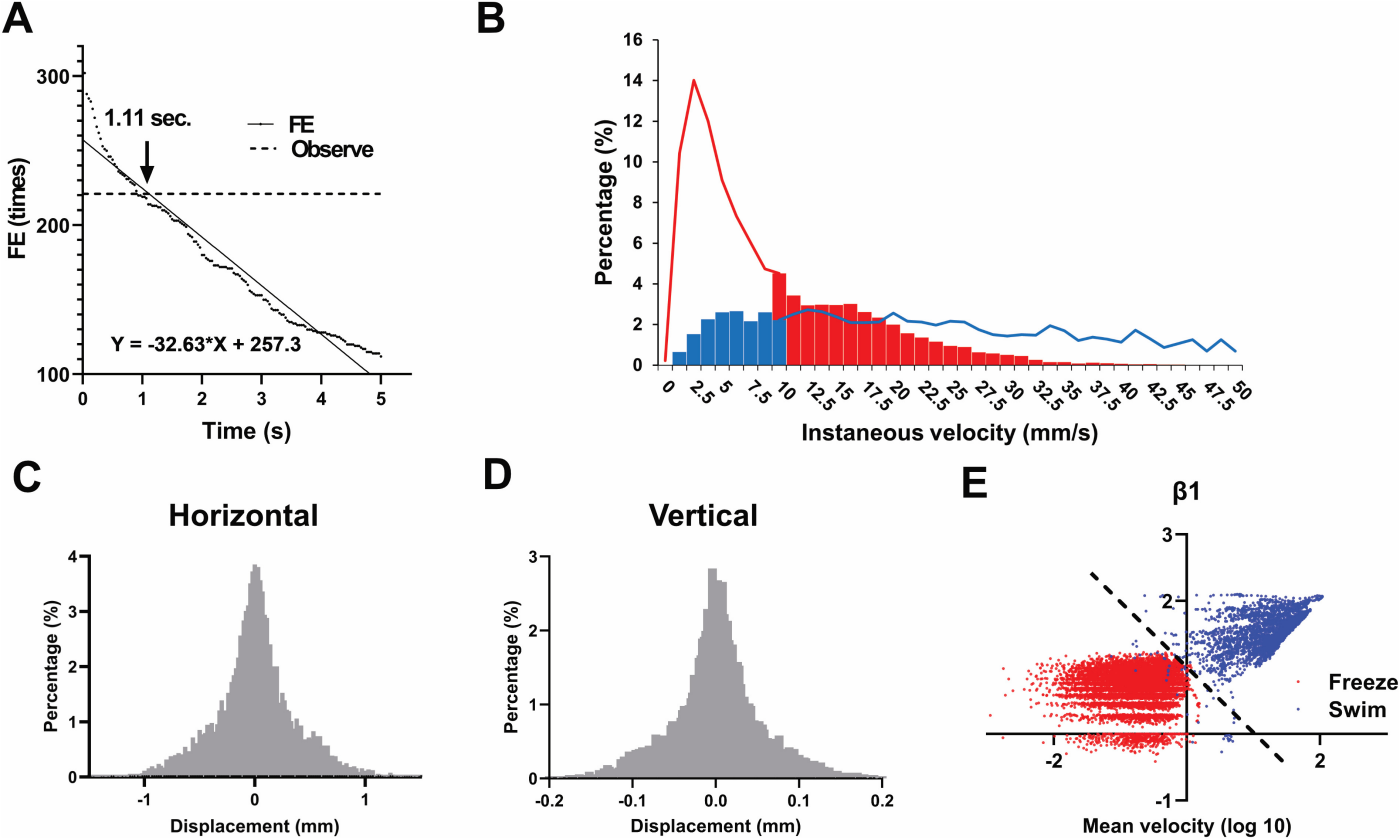
Optimization of entry and freezing detection after tracking. **(A)** Determination of the optimal region-entry time threshold (1.11 s) for defining valid entries into the upper half of the tank based on F-score analysis. This analysis was conducted using 82,887 frames collected from seven females and 98,420 frames from seven male zebrafish. **(B)** Comparison of manual observation and software-based freezing detection. Solid lines and bars indicate manual annotation and software classification, respectively (blue: swimming; red: freezing). **(C,D)** Histogram of horizontal **(C)** and vertical **(D)** displacements during manually confirmed freezing frames, indicating Gaussian-distributed noise. **(E)** Instantaneous and average velocities were plotted on a logarithmic scale. Both clusters can be approximately separated with the axis *y* = *x*. For the analysis in **B–E**, freezing behavior was analyzed using 10,488 frames (in red) collected from one male and one female fish, while swimming behavior was analyzed using 3,217 frames (in blue) collected from three different males and one female fish.

Analysis of zebrafish trajectories during NTT revealed the following: when the detection of entry into the upper region was solely based on the vertical y-coordinate distribution, brief upward excursions, followed by immediate returns to the bottom—as well as fish swimming continuously near the central zone—caused the apparent entry frequency to be overestimated. To address this issue, we defined a parameter as the time a fish remains in a new region after crossing the central *y*-axis line (either from bottom to top or vice versa). We collected video data that included segments wherein entries and exits were ambiguous, and compared the estimated events using this parameter ranging from 0 to 5 s in increments of 1 frame (1/30 s) with the visual annotations of experienced experimenters as the reference. Subsequently, we evaluated each setting and found that an interval of approximately 1.11 s maximized the accuracy (Figure 2A). The F-score exceeded 98% from frame 24 to frame 39. When analyzed separately, the estimated thresholds were 1.03 s for males and 0.78 s for females, indicating only a modest difference between sexes. For all subsequent analyses, FE was determined using a threshold of 1 s for practical reason.

The differences between manual observation and software-based tracking for the detection of freezing behavior are shown in Figure 2B. The solid line represents manual observations, whereas the bar graph indicates the results from software tracking. In the bar graph, blue and red denote periods classified as swimming and freezing, respectively. The red bar regions indicate instances where the fish were actually motionless (freezing); however, as software tracking was based on centroid recognition and frame-wise evaluation, artifacts appeared in the *x*-axis displacement, incorrectly exceeding the threshold of 10 mm/s [because freezing was defined as movement less than 10 mm/s in the software (Audira et al., 2021), as described in section “2.4 Video analysis”]. Conversely, the blue bar regions represent instances where the fish were visually swimming but were classified as freezing by the software owing to direction changes or momentary pauses lasting less than 1 s, leading to a calculated speed below 10 mm/s. The proportions of these misclassifications were 31.6 and 14.5% for the former and latter, respectively, indicating a non-negligible amount of error. After software tracking, frames that were visually confirmed as freezing were extracted, and the displacement per frame was plotted as a histogram. The resulting distribution revealed Gaussian noise (flicker) following a normal distribution (Figures 2C, D).

To prevent these false positives, we compared two approaches—logistic regression and discriminant analysis using the Mahalanobis distance—across multiple parameters (instantaneous velocity, acceleration, mean velocity, heading, etc.). Consequently, we found that logistic regression using the following three parameters was the most suitable. After testing various combinations, we derived a regression equation using only these three parameters that achieved high accuracy. When applied to a new dataset with known outcomes, it consistently attained an F-score above 95%.

Regression equation = 30.447–1.518 β_1_–16.45 β_2_–103.287 β_3_

**β_1_: Log-transformed ratio of instantaneous and average velocities**

In true freezing, screen-flicker noise tends to inflate the sum of instantaneous speeds. Ideally, one would sum the instantaneous speeds over 30 frames (30 fps), divide by the mean speed, and subsequently consider the base-10 logarithm. To reduce the computational cost, we instead used the following formula, wherein the sum over 31 points was multiplied by 30/31 and divided by the mean speed, while considering the base-10 logarithm (Figure 2E):


$$\beta_{1}=l\,o\,g_{10}\,\left\{\frac{30}{31}\,\left(\frac{1}{\bar{v}\,\left(t\right)}\right)\,\overset{15}{\underset{i=-15}{\sum }}v\,\left(t_{i}\right)\right\}$$

**β_2_: Standard deviation of the vertical position**

The use of standard deviations rather than variances improved the model accuracy. While standard deviation is mathematically the square root of variance and thus always increases monotonically with it, in our case, the variance values for vertical position during freezing were often less than one. Under these conditions, the standard deviation becomes numerically larger than the variance, providing a more distinguishable signal for classification. Fish predominantly swim horizontally, making it difficult to distinguish their true movements from tracking noise along that axis. In contrast, vertical movements are infrequent; thus, noise in the vertical component is more readily detectable. Summarizing fluctuations in the vertical trajectory through standard deviation increased the accuracy. We also evaluated the horizontal component but it had no impact on model performance.

**β_3_: Standard deviation of average velocity**

False detections were common when the fish began to swim or came to a halt. Large fluctuations in instantaneous speed created the impression that fish were moving when they were not. To avoid this issue, we calculated the variance of mean speed over 31 consecutive frames (15 before and after the point of interest) and used its standard deviation.

### 3.2 Evaluating the need for pre-test restraint

**FIGURE 3:**
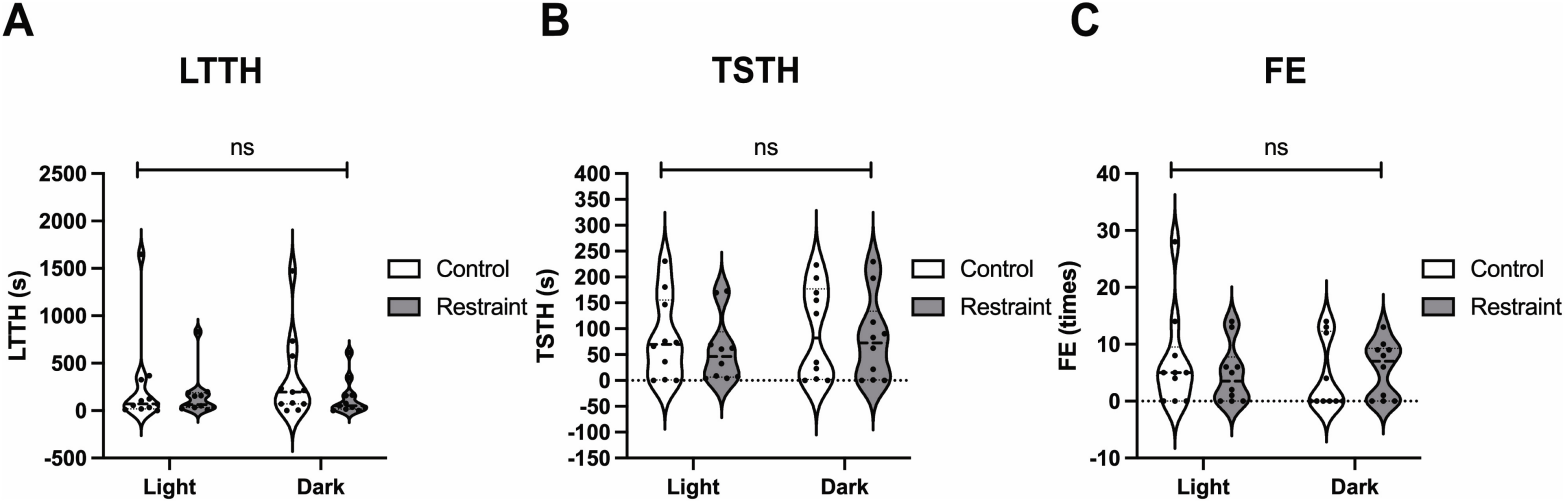
Effects of pre-test stress loading on the outcomes of the NTT. Values of latency to enter the top half (LTTH) **(A)**, time spent in the top half (TSTH) **(B)**, and frequency of entries (FE) **(C)** during the NTT under combinations of light/dark conditions and no-restraint/restraint stress. Although no significant differences were observed in mean LTTH across all conditions, variability was significantly reduced (*p* < 0.05, F-test) under restraint compared with that in the control under both light and dark conditions. The same individuals (*n* = 10; 4 males and 6 females) were used across all conditions.

Although various protocols have been reported for NTT, some include pre-test stress loading (Fontana et al., 2022; Ichikawa et al., 2023). Therefore, we evaluated four stress-loading patterns—restraint, darkness, and their combination—and analyzed their effects on NTT parameters using the same individuals, as shown in Figure 1. The LTTH values were 251.2 ± 144.7 s for the light-control (no restraint), 159.1 ± 70.6 s for the light-restraint, 332.7 ± 134.3 s for the dark-control, and 136.2 ± 56.8 s for the dark-restraint groups (Figure 3A). The TSTH values were 81.1 ± 25.5 s for the light-control, 59.1 ± 20.4 s for the light-restraint, 93.6 ± 28.4 s for the dark-control, and 79.8 ± 25.9 s for the dark-restraint groups (Figure 3B). The FE values were 6.9 ± 2.7 for the light-control, 4.7 ± 1.7 for the light-restraint, 4.3 ± 1.9 for the dark-control, and 5.6 ± 1.6 for the dark-restraint groups (Figure 3C). The results of the F-test are shown in Table 1. No significant differences were observed among the groups in the mean values of LTTH, TSTH, or FE. However, LTTH showed a significant reduction in variability (*p* < 0.05) under the both light and dark conditions when restraint was applied compared with that in the control. In particular, restraint stress was found to suppress variability in the output parameters of NTT. Additionally, under dark conditions, abnormal behaviors were observed during the test, including jumping out of the tank (28.5%). Based on these findings, we decided to apply restraint stress immediately before NTT in the following experiments.

**TABLE 1:** Statistical summary of F-tests for behavioral variability under different pre-test stress conditions in the NTT.

Parameter	Light-control vs. dark-restraint		Light-restraint vs. dark-restraint		Light-control vs. light-restraint		Dark-control vs. dark-restraint	
	F(9, 9)	*p*-value	F(9, 9)	*p*-value	F(9, 9)	*p*-value	F(9, 9)	*p*-value
LTTH	6.184	0.006	0.127	0.003	14.410	0.000	0.295	0.042
TSTH	0.803	0.374	0.620	0.244	1.569	0.256	1.211	0.390
FE	1.937	0.169	1.127	0.431	2.677	0.079	1.557	0.260

### 3.3 Impact of water temperature on NTT

**FIGURE 4:**
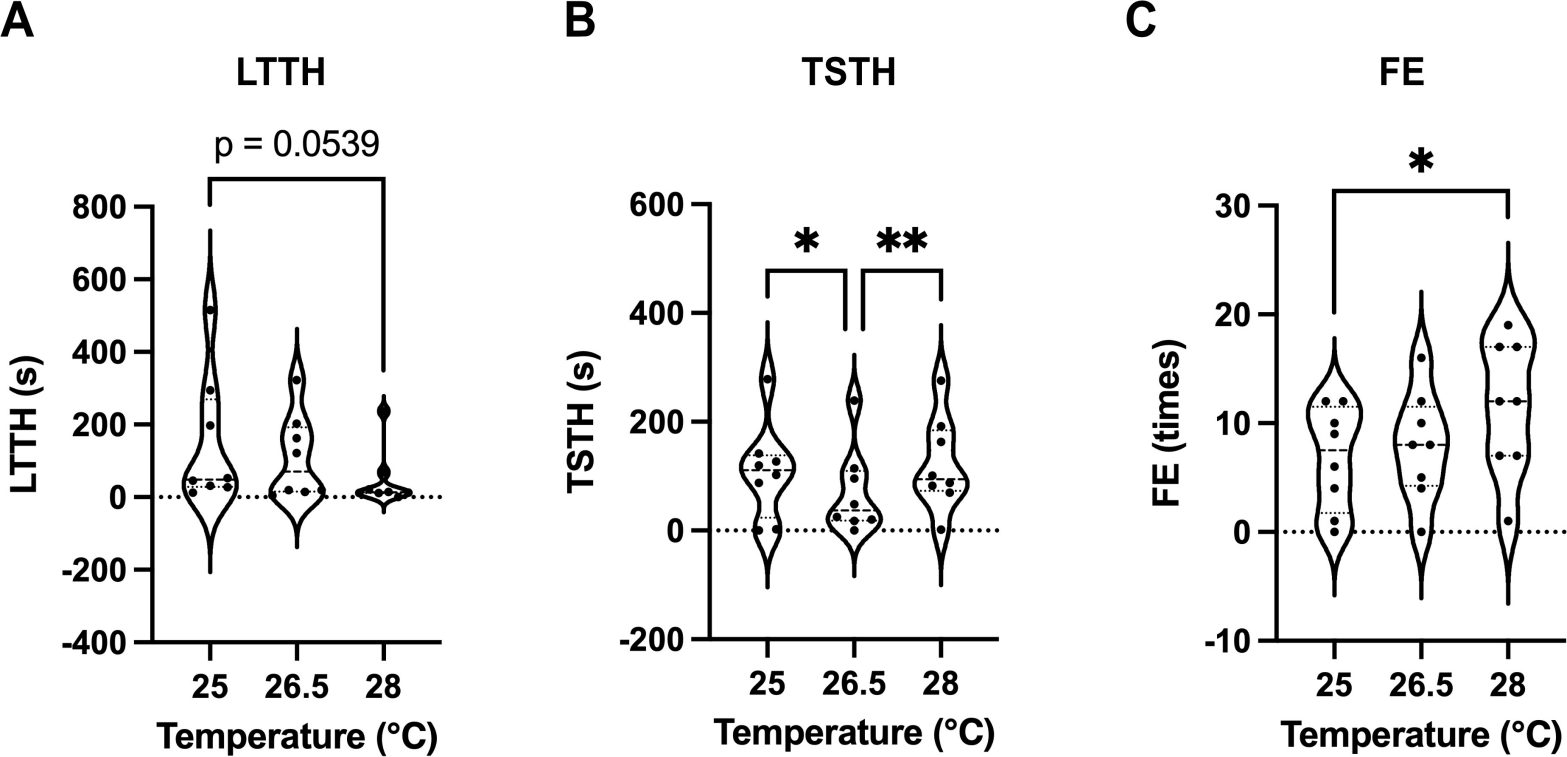
Effects of subtle temperature variations on outcomes of the NTT. **(A–C)** Values of latency to enter the top half (LTTH) **(A)**, time spent in the top half (TSTH) **(B)**, and frequency of entries (FE) **(C)** during NTT at water temperatures of 25, 26.5, and 28 °C. Considering the variability, LTTH at 28 °C was significantly reduced compared with that at 25 °C (*p* < 0.05, F-test). The same individuals (*n* = 8; 4 males and 4 females) were used across all conditions. *p < 0.05, **p < 0.01.

The optimal water temperature for zebrafish is 26 °C, and extreme temperature changes, such as 18 or 34 °C, can affect anxiety-like behavior and novelty-seeking tendencies (Angiulli et al., 2020; Nonnis et al., 2021). We hypothesized that even subtle temperature changes may influence the outcomes of NTT, and thus conducted experiments at temperatures of 25, 26.5 (control), and 28 °C. Water temperatures were strictly regulated, with a maximum deviation of ± 0.1 °C. The LTTH values were 147.1 ± 63.4 s for 25 °C, 108.2 ± 40.8 s for 26.5 °C, and 47.1 ± 28.0 s for 28 °C (Figure 4A). The TSTH values were 107.5 ± 31.0 s for 25 °C, 70.1 ± 28.0 s for 26.8 °C, and 121.6 ± 85.0 s for 28 °C (Figure 4B). The FE values were 6.8 ± 1.7 for 25 °C, 7.9 ± 1.8 for 26.5 °C, and 11.5 ± 2.2 for 28 °C (Figure 4C). The results of the F-test are shown in Table 2. LTTH exhibited a decreasing trend with increasing temperature (Figure 4A). TSTH was significantly higher at both 25 and 28 °C than at 26.5 °C (*p* < 0.05), although no significant difference was observed in TSTH between 25 and 28 °C (Figure 4B). FE significantly increased (*p* < 0.05) with increasing temperature from 25 to 28 °C (Figure 4C). Additionally, the variability in LTTH decreased with increasing temperature, and a significant difference was observed in LTTH between 25 and 28 °C (*p* < 0.05). This variability is attributed to the presence of fish that showed high sensitivity to low temperatures, even at 25 °C, and exhibited reduced activity. Notably, at 28 °C, 62.5% of the fish reached the upper zone of the tank within the first 15 s of NTT, making it difficult to detect subtle differences and thus limiting the applicability of this condition for compound screening. Therefore, 26.5 °C was considered the most appropriate temperature for conducting NTT.

**TABLE 2:** F-test summary for temperature effects on NTT outcomes.

Parameter	25°C vs. 26.5°C		26.5°C vs. 28°C		25°C vs. 28°C	
	F(7, 7)	*p*-value	F(7, 7)	*p*-value	F(7, 7)	*p*-value
LTTH	2.412	0.134	5.110	0.024	2.119	0.172
TSTH	1.229	0.396	0.867	0.428	1.065	0.468
FE	0.911	0.453	0.645	0.289	0.588	0.250

### 3.4 Net-chasing increases the freezing time

**FIGURE 5:**
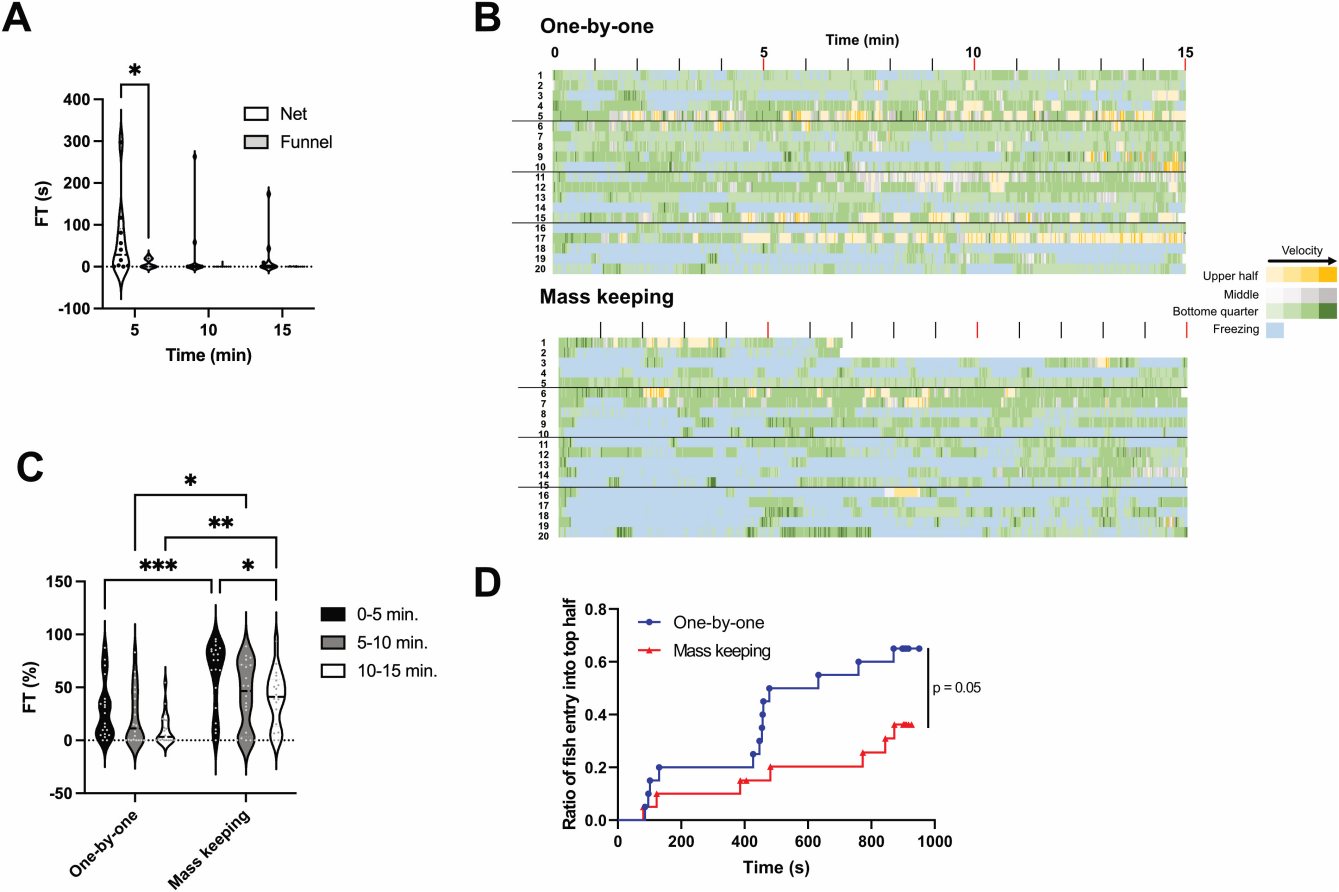
Effects of net-chasing on freezing time and exploratory behavior. (A) Freezing time (FT) measured in 5-min intervals during the novel tank diving test (NTT) using either a net or a funnel for fish transfer. During the first 5 min, FT was significantly higher in the net-transfer group than that in the funnel-transfer group (*p* < 0.05). Variability was significantly greater in the net-transfer group than in the funnel-transfer group across all time points (*p* < 0.05, F-test). The same individuals were used across all conditions (*n* = 10; 5 males and 5 females). *p < 0.05. (B) Heatmaps showing the spatial position and swimming speed of individual fish during a 15-min NTT following either one-by-one or mass breeding conditions. The colors represent the positional zones: yellow, gray, and green represent the upper half, middle half (second and third quartiles from the bottom), and bottom quarter of the tank, respectively. The color intensity reflects the swimming speed, with darker shades indicating faster movement. Freezing episodes are shown in light blue. The *y*-axis represents individual fish. The same population of fish was used in both the one-by-one and mass breeding conditions; however, the individual IDs do not correspond to the same fish across conditions. (C) FT measured in 5-min intervals corresponding to the conditions shown in (B). In all time intervals, FT was significantly higher in the mass breeding group than that in the one-by-one group. *p < 0.05, **p < 0.01, ***p < 0.001. (D) Kaplan–Meier curves showing the cumulative number of fish that entered the upper half of the tank for the first time over the course of the test, corresponding to the conditions in (B). For B–D, the same individuals were used across all conditions (*n* = 20; 10 males and 10 females).

When transferring fish from the breeding tank to a new tank for testing, small nets are generally used. However, there are differences in the experimenter’s technique when using nets. Hence, in this study, we employed a method of transferring fish between tanks using a funnel instead of a net. A comparison between transferring fish to the novel tank using a funnel and net revealed that FT significantly (*p* < 0.05) increased during the first 5 min when the net was used (Figure 5A). As the observation period progressed, FT in the net-transfer group gradually decreased. Variability in the net-transfer group was significantly (*p* < 0.05) greater than that in the funnel-transfer group during the entire observation time. The FT values were 61.4 ± 29.1 s (0–5 min), 32.2 ± 26.3 s (5–10 min) and 22.7 ± 17.3 s (10–15 min) for the net-transfer group, and 33.0 ± 16.9 s (0–5 min), 5.4 ± 4.3 s (5–10 min) and 0.3 ± 0.2 s (10–15 min) for the funnel-transfer group. The results of the F-test are shown in Table 3.

**TABLE 3:** F-test summary for net-chasing effects on FT.

**Net vs. funnel**					
**5 min**		**10 min**		**15 min**	
**F(7, 7)**	***p*-value**	**F(7, 7)**	***p*-value**	**F(7, 7)**	***p*-value**
0.014	0.000	0.002	0.000	0.000	0.000

**Time-cource**						
**Condition**	**5 min vs. 10 min**		**10 min vs. 15 min**		**5 min vs. 15 min**	
	**F(7, 7)**	***p*-value**	**F(7, 7)**	***p*-value**	**F(7, 7)**	***p*-value**
Net	1.223	0.385	2.304	0.115	2.817	0.069
Funnel	8.068	0.002	50.568	0.000	407.989	0.000

To better reflect actual experimental conditions, we conducted a comparative experiment between two handling methods: (1) the “mass keeping” condition, wherein 20 fish were group-housed in a 2-L tank and individually transferred to the acclimation tank using a net; and (2) the “one-by-one” condition, wherein fish were individually housed from the previous day and transferred using a funnel. As shown in Figure 5B, under the mass breeding condition (lower panel), the duration of freezing behavior (indicated in blue) was longer than that under the one-by-one condition. Furthermore, the extent of the blue area increased with increasing number of transferred fish. Figure 5C shows the cumulative FT measured at 5-min intervals. The FT values were 28.2 ± 6.1 s (0–5 min), 22.0 ± 5.9 s (5–10 min) and 10.6 ± 3.3 s (10–15 min) for the one-by-one group, and 62.4 ± 7.1 s (0–5 min), 42.1 ± 7.1 s (5–10 min) and 39.6 ± 6.2 s (10–15 min) for the mass breeding group. The results of the F-test are shown in Table 4. Between 0–5, 5–10, and 10–15 min, the mass breeding group exhibited significantly greater FT compared with the one-by-one group (*p* < 0.05). Additionally, in the mass breeding group, FT gradually decreased over time.

**TABLE 4:** F-test summary for FT across 5-min intervals with net-chasing conditions.

**Time-cource**						
**Condition**	**0–5 min vs. 5–10 min**		**5–10 min vs. 10–15 min**		**0–5 min vs. 10–15 min**	
	**F(19, 19)**	***p*-value**	**F(19, 17)**	***p*-value**	**F(19, 17)**	***p*-value**
One-by-one	1.066	0.555	3.248	0.007	3.462	0.995
Mass breeding	0.994	0.505	1.463	0.218	1.471	0.214

**One-by-one vs. mass breeding**		
**Time-course**	**F(19, 19)**	***p*-value**
0–5 min	0.731	0.749
5–10 min	0.682	0.794
10–15 min	0.992*	0.008

The asterisk (*) is used only for the 10–15 min time point under the One-by-one condition to indicate that the value corresponds to F(19,17), whereas all other conditions are F(19,19).

The number of fish that experienced entering the upper half of the tank at least once increased with the progression of NTT. The rate of increase was significantly (*p* < 0.05) lower in the mass breeding group compared with that in the one-by-one group (Figure 5D). Moreover, in the one-by-one group, the number of fish reaching the upper half began to increase at approximately 400 s; however, this phenomenon was not observed in the mass breeding group.

### 3.5 NTT is unaffected by the learning abilities of zebrafish

**FIGURE 6:**
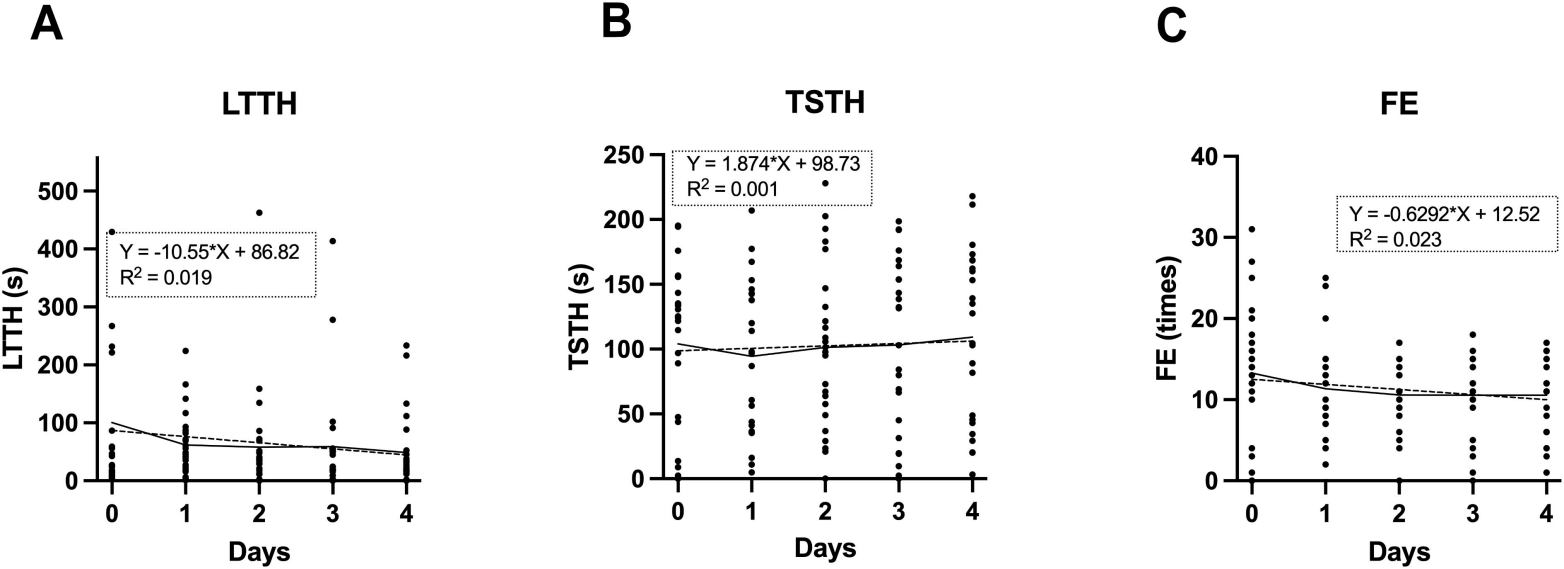
Assessment of learning effects during repeated NTT trials. **(A–C)** Values of latency to enter the top half (LTTH) **(A)**, time spent in the top half (TSTH) **(B)**, and frequency of entries (FE) **(C)** measured across five consecutive days of NTT. The same individuals were used across all conditions (*n* = 24; 13 males and 11 female). Simple linear regression analysis was conducted, and regression lines are indicated by dotted lines.

Zebrafish are also used as models in learning tests, such as fear conditioning (Fu et al., 2025; Martin and Lovett-Barron, 2025) and avoidance learning (Yang et al., 2018). To examine whether a similar learning effect occurs in NTT, we conducted the test on the same individuals for five consecutive days and evaluated three parameters. Based on the findings presented in Figures 3, 4, all experiments were conducted under the following standardized conditions: 26.5 °C, bright lighting, and restraint. As shown in Figure 6, LTTH, TSTH and FE did not exhibit significant trends over the days. In fact, coefficients of determination (R^2^) were all approximately 0.2 or lower. These results suggest that repeated daily exposure to the NTT had minimal influence on these behavioral parameters.

### 3.6 FE follows a Poisson process

**FIGURE 7:**
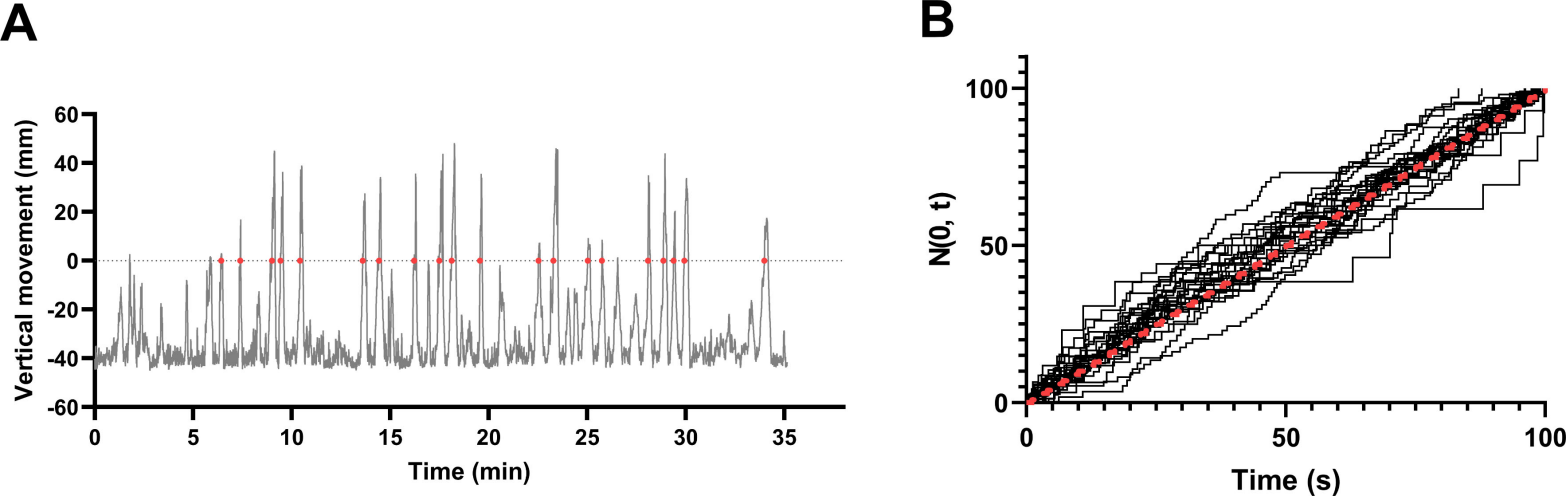
Entry events into the upper half of the tank follow a Poisson process. **(A)** Vertical movement of individual male fish plotted over time. Red dots indicate events identified as entries into the upper half of the tank. **(B)** Cumulative standardized entry events based on the vertical movement data from 38 fish (19 males and 19 female). The red dashed line represents the expected values under a Poisson process. Most individuals closely followed the expected distribution.

When visualizing the vertical movement of fish, variability was observed in the pattern of the graph up to the third vertical entry event (FE), including the initial LTTH. However, beyond the third entry into the upper zone, the intervals between successive events appeared to follow a more consistent pattern (Figure 7A). Therefore, data obtained after the first 5 min of NTT were standardized and assessed for conformity to a Poisson process. As shown in Figure 7B, few observed values (dashed line) deviated from the expected values (solid line), indicating that the data followed a Poisson process.

## 4 Discussion

### 4.1 Refinement of parameter calculation after tracking

As demonstrated in previous studies (e.g., Assad et al., 2020; Audira et al., 2021), LTTH is a rational and widely accepted indicator of anxiety-like behavior. However, simply counting the number of entries into the upper half of the tank based solely on the *y*-coordinate can lead to overestimation. This occurs when distinct transient behaviors, such as briefly surfacing and immediately descending, or repetitive swimming near the boundary line, are erroneously counted as multiple entries. The former is characterized by large vertical and minimal horizontal movement, whereas the latter shows the opposite pattern. However, vertical or horizontal movement alone is not suitable for accurately detecting entry into a designated region. Instead, adjusting the threshold based on dwell time after crossing the boundary is more appropriate. A comparison between automated classification and human visual assessment indicated that a threshold of 1.11 s yielded the best agreement (Figure 2A).

FT is another commonly used behavioral index for anxiety and fear in zebrafish (Pluimer et al., 2023; Masuda et al., 2024). The selection of a 10 mm/s threshold for defining freezing behavior was based on prior studies using similar criteria for zebrafish locomotion analysis (Audira et al., 2021). Although this can be easily verified through visual inspection of video footage, relying solely on numerical data often results in false positives (Figure 2B). We recognize that adjusting this threshold can significantly affect classification outcomes: a lower threshold (e.g., < 5 mm/s) might reduce false positives caused by minor tracking fluctuations, but may also increase false negatives by failing to capture brief episodes of true immobility. Given this trade-off, we prioritized consistency with established protocols while minimizing classification bias.

Automated classification based solely on numerical data is prone to non-negligible misclassification. False negatives often arise from accumulated tracking artifacts, particularly frictional noise, which can distort the identification of freezing behavior. The trajectory data display small random fluctuations in both vertical and horizontal directions, which reflect Gaussian noise and can typically be reduced through smoothing (Figures 2C, D). False positives may also occur, especially during brief exploratory or directional movements, and are more pronounced in stationary fish, where minor tracking noise is mistaken for motion. In contrast, such noise has minimal impact when tracking actively moving individuals. However, excessive smoothing may obscure meaningful behavioral signals and inadvertently increase false positives. To address these challenges, we applied logistic regression using three key parameters (β_1_, β_2_, and β_3_), which resulted in a classification outcome that matched manual observation with 95% agreement.

The parameter β_1_ enables approximate classification and is highly robust (Figure 2E). This index captures differences in instantaneous velocity between active swimming and freezing states: the former is characterized by a mixture of large and small movements, whereas the latter follows a Gaussian distribution. In contrast, β_2_ offers high classification accuracy but owing to its very small value, it is sensitive to outliers. Since zebrafish predominantly swim in horizontal directions (left or right), horizontal displacement often fails to differentiate true movement from mechanical noise. As a result, vertical fluctuations are more suitable for detecting such noise. The parameter β_3_ exhibits characteristics intermediate between β_1_ and β_2_. It appears to reflect fluctuations in mean velocity during directional changes, which may contribute to the misclassification of freezing behavior. Although the training data were classified cleanly as if only β_2_ and β_3_ were used, performance on new data revealed variability, with some cases being classified highly accurately and others less so. The proposed model uses three parameters (β_1_–β_3_). Increasing the number of parameters could potentially improve the accuracy; however, this can also raise concerns regarding overfitting and increase computational demands. Considering the processing capacity of standard personal computers, the use of three parameters represents a practical and appropriate balance.

### 4.2 Pre-test stress loading by restraint

Exposure to novel environments, such as being transferred to a new tank, serves as an anxiety-inducing stimulus and is the foundational basis for NTT as an assay for anxiety-like behavior. In mammalian models, various environmental stressors can trigger anxiety-related behaviors (Walf and Frye, 2007; Milner and Crabbe, 2008; La-Vu et al., 2020). In zebrafish, two commonly used stress inducers are darkness (Haney et al., 2020) and physical restraint (Ghisleni et al., 2012). In this study, we explored whether combining these stressors with NTT could lead to a more refined and consistent behavioral protocol (Figure 1). As shown in Figure 3, no significant differences were observed among groups in the mean values of the three key parameters, namely, LTTH, TSTH, and FE. However, within the light and dark conditions, variability in LTTH was significantly (*p* < 0.05) reduced. Although the combination of restraint stress with NTT has been used by some scholars, to the best of our knowledge, no reports have rigorously examined its effects. This method may have been adopted based on empirical observations suggesting that it helps reduce variability in the output parameters of NTT. Under the combination of darkness and restraint, a high proportion of fish (28.5%) attempted to jump out of the tank during the experiment. Temporary darkness represents an unnatural stimulus for zebrafish, as such conditions do not typically occur in their natural environment. The abrupt transition between light and dark may have elicited complex neural responses, potentially leading to more complicated and variable behavioral outcomes.

Reduction in variability under both restraint conditions, some individuals still exhibited near-zero values for LTTH, TSTH, and FE. These may reflect intrinsic individual differences in stress responsivity that are not fully mitigated by pre-test conditioning. Nevertheless, we identified restraint under light conditions as the most suitable pre-test stress protocol based on its balance of reduced behavioral variability, absence of extreme reactions (e.g., escape-like jumps observed under dark conditions), and ecological validity. The light-restraint condition avoids the artificiality and heightened unpredictability associated with sudden darkness, while providing sufficient stress loading to stabilize behavioral outputs across trials. This optimized protocol contributes to improved reproducibility and interpretability in NTT-based assessments of anxiety-like behavior.

### 4.3 Effects of subtle temperature variations and net-chasing

Although NTT is a relatively simple behavioral assay, issues with reproducibility are not uncommon. In this study, we used zebrafish individually labeled with elastomer tags to investigate the influence of subtle temperature changes on behavioral outcomes (Figure 4). LTTH exhibited a decreasing trend with increasing temperature (*p* < 0.1), while FE increased significantly (*p* < 0.05). Our results revealed that even minor temperature variations—within a range of 3 °C—affected the variability of key parameters such as LTTH and FE, contrary to previous assumptions (Ghisleni et al., 2012; Nonnis et al., 2021). These findings highlight the necessity of conducting NTT in a dedicated, temperature-controlled environment with precisely regulated room and water temperatures.

Most scholars transfer test fish between tanks using nets; however, our findings demonstrated that this procedure can significantly impact behavior (Figure 5). In particular, we found that fish collected by net-chasing exhibited increased FT compared with those gently transferred using a funnel with water. This effect is likely attributable to physical stress, although the potential contribution of chemical alarm substances cannot be entirely ruled out. While ostariopterin and daniol sulfate have been identified in zebrafish skin extracts as candidate alarm substances (Masuda et al., 2024), it remains unclear whether these compounds are actually released during net-chasing or capture. Although LTTH reduced in the mass keeping group (affected by the accumulated net-chasing effect) (Figure 5C), this reduction seemed to be a secondary effect of excessive FT rather than a true anxiolytic outcome. In particular, fear induced by net-chasing may be misinterpreted as anxiety-like behavior. As elevated FT can influence other behavioral parameters, NTT trials exhibiting abnormally high FT should be considered for repetition to ensure data integrity. Additionally, fish collection and transfer through net-chasing are influenced by the individual experimenter’s skill level, which could further affect the reproducibility of NTT results.

### 4.4 Repeated testing and novelty perception

When NTT is used for applications such as drug testing, it may become necessary to conduct the test on consecutive days. In such cases, the learning ability of zebrafish becomes an important consideration. Previous studies have shown that consecutive days of active avoidance training improve learning ability and lead to the formation of long-term memory in zebrafish (Morin et al., 2013; Reemst et al., 2023). In Figure 6, we observed that daily exposure to the same NTT environment over five consecutive days did not result significant changes in behavioral measures (LTTH, TSTH, and FE), suggesting minimal habituation. This may indicate that zebrafish do not fully perceive the tank as less novel with repeated exposures, or alternatively, that their responses to the context remain stable over time. These findings imply the potential feasibility of using the same individuals for repeated NTT trials, provided that testing is limited to once per day. The results also indicate that intervals of at least one day between trials appear to minimize carry-over effects from the previous trial on the subsequent one.

### 4.5 Estimating the appropriate duration for NTT based on the Poisson process

In this study, we found that the frequency of entries into the upper half of the tank (FE) conforms to a Poisson process. The number of entries per unit time can be interpreted as the event intensity, owing to the independence of events, and the interval between events does not reflects the latency to the previous entry, consistent with the memoryless property. Although slight deviations were observed in the initial FE events—possibly reflecting anxiety-induced stress responses—these discrepancies diminished after approximately three events, and the pattern began to conform to the Poisson model. Although determining the exact timing of this transition is challenging, we adopted 10 min as a practical threshold. In particular, we defined the first 5 min as the stress-response period, the interval from 5 to 10 min as the transition phase, and the period after 10 min as the steady state. Data collected after the 10-min mark clearly followed the Poisson process (Figure 7), supporting the validity of this framework. Although the recording durations of NTT have varied widely in previous studies—from as short as 2 min (Ichikawa et al., 2023) to as long as 1 h (Yoshida, 2022)—our findings suggest that a recording time of at least 5 to 10 min is sufficient to capture behavior representative of anxiety toward a novel environment.

## 5 Conclusion

NTT is a widely used behavioral assay that enables the quantitative analysis of anxiety-like behaviors in small fish species, such as zebrafish. It enables the assessment of psychological effects of anxiety-inducing or anxiolytic compounds that are not readily observable through external appearance. Owing to its simplicity, NTT has been adopted by many scholars; however, its interpretation and application remain subjects of debate. A standardized protocol has yet to be established, and issues with reproducibility are frequently encountered. In this study, we aimed to reduce variability in NTT data, including individual differences among subjects. To achieve this, we propose several methodological refinements: a standardized data processing workflow following animal tracking, the application of pre-test restraint stress, strict temperature control, and use of a funnel instead of a net to avoid net-chasing stress. Collectively, these improvements possibly represent the most refined and reliable version of the NTT protocol.

## Data Availability

The raw data supporting the conclusions of this article will be made available by the authors, without undue reservation.
