# Supplementary material for: Refinement of the novel tank diving test: toward standardized and robust analysis of anxiety-like behavior in zebrafish
Source: Front Behav Neurosci. 2025 Oct 29;19:1624277. doi: 10.3389/fnbeh.2025.1624277 (PMC12605500; doi:10.3389/fnbeh.2025.1624277)
Supplement: Supplementary file 1 [file Data_Sheet_1.docx]

**Supplementary Methods**

1. **Handling of water used in experiments**

Water for each test was pre-heated and aerated in large plastic containers (maximum capacity: ~60 L). The desired temperature was maintained using an aquarium heater and monitored with an electronic aquarium thermometer. Based on prior experience, water temperature typically dropped by 0.1–2 °C during transfer; therefore, heaters were set slightly above the target temperature to compensate. These containers were used exclusively for this purpose to ensure cleanliness.

All equipment used for uncontaminated water was strictly separated from that used for fish handling. Equipment involved in pre-test stress procedures and experimental tanks was thoroughly washed and rinsed with distilled water before each trial.

To ensure consistent conditions, uncontaminated water was always used. Specifically, when transferring fish from the acclimation tank to the restraint tank, water from the acclimation tank was diluted twofold by mixing with an equal volume of fresh water.

1. **Evaluating pre-test stress variations (Results section 3.2)**

Each fish was individually identified. Among the four stressor combinations, the light / no-restraint condition was designated as the control and always tested first to avoid potential carry-over effects. The remaining three conditions were tested using a full factorial design (3! = 6 orders), with two fish per order (total = 12). However, data from two individuals were lost due to recording errors and escapes.

For the no-restraint condition, fish were transferred with the surrounding water by tilting the tank, using overflow to prevent escape—without any use of nets or funnels. A 72-hour interval was maintained between tests, during which fish were group-housed under standard conditions.

1. **Water temperature conditions (Results section 3.3)**

To stabilize testing conditions, the experiment room was air-conditioned starting the previous night. Fish were transferred to this room to equilibrate tank water temperature with the ambient environment. Since precise temperature control was critical and potential system failures could occur, a randomized block design was not used. Instead, tests were conducted in the following order: control → high temperature → low temperature, as high temperature was expected to have a milder impact than low temperature based on preliminary observations.

1. **Detailed protocol for net-chasing tests (Results section 3.4)**

This investigation consisted of two independent experiments using different individuals, both examining the impact of net-chasing.

- First experiment: A basic comparison.
- Second experiment: Focused on the extent of net-chasing effects, including indirect effects on cohabiting fish.

We hypothesized that net-chasing would affect not only the focal fish but also others sharing the same water, possibly through alarm substances known to influence freezing behavior (Matsuda, 2024). Accordingly, water management was carefully controlled.

Experimental group:

Twenty fish were housed overnight in a single breeding system tank (2 L total volume). No water changes were made after the start of the experiment, allowing net-chasing effects to accumulate. Fish were tested one-by-one, and those tested later were expected to experience greater influence.

Control group:

Each fish was isolated in a 300 mL plastic cup overnight. To reduce contamination from stress-related secretions, cups were diluted by adding an equal volume of fresh water three hours after initial transfer (after acclimation), followed by careful pouring to retain 300 mL.

The same twenty fish were used for both experiments. In the shared tank condition, fish were randomly scooped without selecting specific individuals to avoid additional behavioral effects. Therefore, individual test order was not matched between experiments.
